# Perinatal risk factors for type 1 diabetes revisited: a population-based register study

**DOI:** 10.1007/s00125-019-4874-5

**Published:** 2019-04-30

**Authors:** Ingeborg Waernbaum, Gisela Dahlquist, Torbjörn Lind

**Affiliations:** 1 Department of Statistics, USBE, Umeå University, Umeå, Sweden; 2 Paediatrics, Department of Clinical Sciences, Umeå University, SE-901 85 Umeå, Sweden

**Keywords:** Birthweight, Case–control study, Diabetes mellitus type 1, Perinatal risk factors, Urinary tract infection

## Abstract

**Aims/hypothesis:**

Single-centre studies and meta-analyses have found diverging results as to which early life factors affect the risk of type 1 diabetes during childhood. We wanted to use a large, nationwide, prospective database to further clarify and analyse the associations between perinatal factors and the subsequent risk for childhood-onset type 1 diabetes using a case–control design.

**Methods:**

The Swedish Childhood Diabetes Register was linked to the Swedish Medical Birth Register and National Patient Register, and 14,949 cases with type 1 diabetes onset at ages 0–14 years were compared with 55,712 matched controls born from the start of the Medical Birth Register in 1973 to 2013. After excluding confounders (i.e. children multiple births, those whose mother had maternal diabetes and those with a non-Nordic mother), we used conditional logistic regression analyses to determine risk factors for childhood-onset type 1 diabetes. We used WHO ICD codes for child and maternal diagnoses.

**Results:**

In multivariate analysis, there were small but statistically significant associations between higher birthweight *z* score (OR 1.08, 95% CI 1.06, 1.10), delivery by Caesarean section (OR 1.08, 95% CI 1.02, 1.15), premature rupture of membranes (OR 1.08, 95% CI 1.01, 1.16) and maternal urinary tract infection during pregnancy (OR 1.39, 95% CI 1.04, 1.86) and the subsequent risk of childhood-onset type 1 diabetes. Birth before 32 weeks of gestation was associated with a lower risk of childhood-onset type 1 diabetes compared with full-term infants (OR 0.54, 95% CI 0.38, 0.76), whereas birth between 32 and 36 weeks’ gestation was associated with a higher risk (OR 1.24, 95% CI 1.14, 1.35). In subgroup analyses (birth years 1992–2013), maternal obesity was independently associated with subsequent type 1 diabetes in the children (OR 1.27, 95% CI 1.15, 1.41) and rendered the association with Caesarean section non-significant. In contrast to previous studies, we found no association of childhood-onset type 1 diabetes with maternal–child blood-group incompatibility, maternal pre-eclampsia, perinatal infections or treatment of the newborn with phototherapy for neonatal jaundice. The proportion of children with neonatal jaundice was significantly higher in the 1973–1982 birth cohort compared with later cohorts.

**Conclusions/interpretation:**

Perinatal factors make small but statistically significant contributions to the overall risk of childhood-onset type 1 diabetes. Some of these risk factors, such as maternal obesity, may be amendable with improved antenatal care. Better perinatal practices may have affected some previously noted risk factors over time.

**Electronic supplementary material:**

The online version of this article (10.1007/s00125-019-4874-5) contains peer-reviewed but unedited supplementary material, which is available to authorised users.

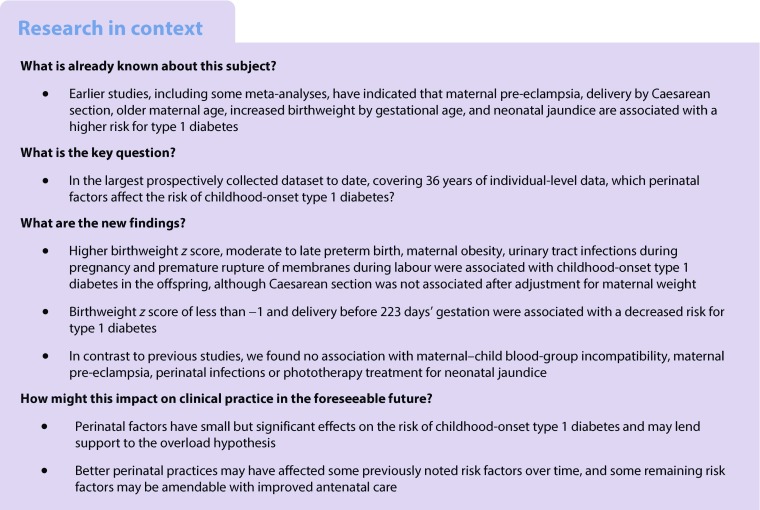



## Introduction

Studies on the natural history of childhood-onset type 1 diabetes mellitus show that markers of beta cell autoimmunity appear several years before the onset of clinical disease, with islet autoantibodies being detectable during infancy [1–3]. Persistent positivity to multiple such autoantibodies is strongly associated with clinical disease [4]. It is therefore of interest to search for exposures that may start the process early in life, leading to type 1 diabetes later during childhood. An early suggestion was that intrauterine viral infections may be important, since the prospective follow-up of a cohort of individuals with rubella embryopathy showed they had a dramatically increased risk of diabetes later in life [5]. Since 1974, all girls (and later all children) in Sweden have been vaccinated against rubella and the disease has become exceedingly rare; the last domestic case of rubella embryopathy was reported in 1985 [6]. Still, the reported incidence of childhood-onset type 1 diabetes in Sweden doubled in the 30 years from 1978 to 2007 [7].

A series of population-based studies originating from prospectively recorded perinatal data from either registries or hospital records have suggested associations between the risk of type 1 diabetes and factors such as maternal pre-eclampsia, delivery by Caesarean section, older maternal age, increased birthweight by gestational age and neonatal jaundice induced by maternal–fetal blood-group incompatibility [8–13]. Some of these have been confirmed in meta-analyses [14–17], whereas others remain controversial [18,19]. Socioeconomic factors may confound some of these risk factors.

The Swedish Childhood Diabetes Register (SCDR) has recorded virtually all incident cases of childhood-onset type 1 diabetes in Sweden since 1 July 1977 [7]. In the current study, we matched four controls to each case in the register and linked data from official health registers, providing a larger database than that used by many meta-analyses, as well as individual-level information on a number of health and socioeconomic exposures. Compared with our own previous analyses of perinatal risk factors for type 1 diabetes using data from the SCDR [8–11], the current study uses around five times as many cases covering a large timespan. Thus, we aimed to use this substantially larger database to conduct an in-depth study of early factors influencing the trajectory of type 1 diabetes development.

## Methods

Our study population consisted of incident type 1 diabetes cases from the SCDR from 1 January 1978 to 31 December 2013 and four controls per case from the Swedish Total Population Register, matched for age (birth year and month) and municipality at the time of diagnosis of the incident case. Statistics Sweden, Stockholm, performed the matching.

For cases and controls, we extracted data from the Swedish Medical Birth Register (MBR) on the type of delivery (vaginal or Caesarean section), the age of the mother at the birth of the child, and the weight, gestational length and gestational age of the child at birth. Furthermore, we obtained data on maternal illnesses (i.e. diabetes, hypertension, pre-eclampsia, eclampsia, urinary tract infections [UTIs], premature rupture of membranes [PROM], pyrexia and other infections during labour). The MBR has recorded data on prenatal, delivery and neonatal care since 1973 [20, 21], and it is mandatory for all healthcare providers to report to the register. Hence, we discharged those born before 1973 (*n* = 13,905). The MBR has been validated and found to be a valuable source of information for research on perinatal events [21]. Very little systematic error was found, and missing data in the register affects prevalence estimates but has little effect on risk estimates. We also collected data from the National Patient Register (NPR) [22], which includes all inpatient diagnoses since 1987 [23]. The register uses the WHO ICD coding system [24] (see Electronic Supplementary Material [ESM] Table 1). The combined use of the MBR and the NPR was suggested from the validation of the MBR in order to decrease problems with missing data for infant diagnoses [21]. From both the MBR and the NPR [22], we collected data on neonatal jaundice, intrauterine and birth asphyxia and respiratory distress of the newborn, neonatal aspiration syndromes, various neonatal infections and whether the child had been treated with phototherapy for neonatal jaundice.

From 1992, data in the MBR on first-trimester maternal height and weight was complete enough to allow calculation and classification of maternal BMI [21]. Normal weight was classified as BMI less than 25 kg/m^2^, overweight as BMI 25–30 kg/m^2^ and obesity as BMI more than 30 kg/m^2^.

To limit the presence of known confounders we restricted our study population to singleton births, children of non-diabetic mothers and children of mothers born in the Nordic countries. The number of individuals in the study population by the mother’s country of birth is displayed in ESM Table 2. We conducted univariate and multivariate analyses for various factors and the risk of type 1 diabetes using conditional logistic regression models, thus considering the matched case–control design. For the univariate analyses of perinatal variables and diagnoses, we also stratified the data by sex and age at diabetes onset. Assuming that the OR approximates the RR, we estimated interactions on an additive scale by calculating the relative excess risk due to interaction (RERI) between sex and the perinatal variables [25–27]. This was not feasible for the interaction between age at onset and diagnoses, since age at onset was a matching variable in the initial case–control design. To avoid bias when assessing the total effect of each risk factor, we use directed acyclic graphs as tools for confounder selection in the multivariate analyses [28–30]. We illustrated the assumptions of underlying mechanisms by causal models in directed acyclic graphs and selected confounders accordingly [31].

Premature birth was defined as delivery before 259 days of gestation, which was then subclassified into moderate to late preterm (224–258 days’ gestation) and very preterm (<224 days’ gestation) [32]. We followed the WHO’s definition of the perinatal period as commencing at 154 days’ gestation and ending at 7 completed days after birth. In our context, the perinatal period contains both maternal and infant variables. Neonatal was defined as relating to or affecting the newborn during the first month of life. Birthweight *z* scores were calculated from intrauterine growth charts [33]. Furthermore, we stratified the birthweight *z* scores to compare ORs between different categories of birthweight *z* scores. Analyses were made by excluding missing variables; however, there were generally few missing variables, except for maternal BMI (ESM Table 3).

To evaluate possible changes in risk factors over time, we also conducted stratified analyses for four different birth cohorts (birth years 1973–1982, 1983–1992, 1993–2002 and 2003–2013). Three different WHO ICD coding systems [24] were used during the study period: ICD 8 (until 1986; http://www.wolfbane.com/icd/icd8.htm), including 4921 cases and 18,739 controls; ICD 9 (1987–1996; www.icd9data.com/2007/Volume1), including 5535 cases and 20,478 controls; and ICD 10 (from 1997; http://apps.who.int/classifications/icd10/browse/2016/en), including 4493 cases and 16,497 controls. In addition, we investigated the association between risk factors and age of onset of type 1 diabetes mellitus.

All analyses were performed using the statistical software R, version 3.4.2 [34] with the package ‘survival’ and the logit function for conditional logistic regression. For the RERI analyses we used bootstrap [26] to calculate CIs using the R package ‘boot’. Collection of matched controls and data linkage were performed at Statistics Sweden and only coded data were delivered back to the research group. The study was approved by the Regional Ethics Review Board at Umeå University and the ethics committee at Statistics Sweden, Stockholm, according to Swedish law on research ethics, and performed in accordance with the Declaration of Helsinki.

## Results

After excluding twins, children born to mothers with diabetes and children with mothers born outside of the Nordic countries, we analysed data from 14,949 cases and 55,712 controls (Table 1). There were 34,095 females (7024 cases) and 36,566 males (7925 cases), and the mean age at diagnosis of type 1 diabetes was 8.29 ± 3.87 years.

**Table 1:** Study population selection and number of individuals related to inclusion/exclusion criteria

Study population, *n*	Cases	Controls
Individuals from the SCDR born 1973–2013	18,766	74,990
Individuals with missing information from the MBR	2345	11,562
Twins	327	1535
Children of diabetic mothers	318	278
Children of non-Nordic mothers	827	5903
Total sample	14,949	55,712

### Maternal factors

In the univariate analyses, there were small but statistically significant increases in the odds for developing type 1 diabetes relative to the referent for being born by Caesarean section and having an older mother (Table 2). The referents for Caesarean section and maternal age were being born by vaginal delivery and for maternal age a unit difference in age, respectively. Pre-eclampsia and eclampsia were not linked to an increased risk of type 1 diabetes (Table 3). Stratification for age at onset of type 1 diabetes did not change these relationships (Table 4). Maternal UTI and PROM were, however, both associated with a higher risk of type 1 diabetes (Table 3).

**Table 2:** Descriptive data for the perinatal variables for cases of childhood-onset type 1 diabetes and controls, and univariate ORs and 95% CIs, adjusted for the case–control design

Variable	Cases	Controls	OR (95% CI)
Overall			
Caesarean section	1853/14,948 (0.12)	6303/55,707 (0.11)	1.11 (1.05, 1.18)
Birthweight (g)	3563 ± 538	3550 ± 549	1.00 (1.00, 1.00)
Gestational age (days)	279 ± 12	279 ± 12	0.99 (0.99, 0.99)
Gestational length^a^			
Very preterm	40/14,922 (0.0027)	269/55,615 (0.0048)	0.56 (0.40, 0.79)
Moderate to late preterm	845/14,922 (0.0566)	2530/55,615 (0.0455)	1.26 (1.16, 1.37)
Full term	14,037/14,922 (0.941)	52,816/55,615 (0.950)	–
Age of mother (years)	28.60 ± 5.12	28.32 ± 5.10	1.01 (1.01, 1.01)
Birthweight *z* score	0.077 ± 1.08	–0.017 ± 1.08	1.08 (1.06, 1.10)
Boys			
Caesarean section	1007/7925	3332/28,638	1.11 (1.01, 1.22)
Birthweight (g)	3626 ± 547	3615 ± 555	1.00 (1.00, 1.00)
Gestational age (days)	279 ± 12	280 ± 12	0.99 (0.99, 1.00)
Gestational length^a^			
Very preterm	19/7908 (0.002)	132/28,592 (0.0046)	0.39 (0.22, 0.69)
Moderate to late preterm	458/7908 (0.058)	1368/28,592 (0.048)	1.17 (1.02, 1.33)
Full term	7431/7908 (0.940)	27,277/28,592 (0.954)	–
Age of mother (years)	28.66 ± 5.11	28.36 ± 5.11	1.01 (1.00, 1.02)
Birthweight *z* score	0.23 ± 1.08	0.13 ± 1.07	1.08 (1.05, 1.10)
Girls			
Caesarean section	846/7023	2971/27,069	1.08 (0.98, 1.20)
Birthweight (g)	3492 ± 519	3483 ± 535	1.00 (1.00, 1.00)
Gestational age (days)	279 ± 12	279 ± 12	0.99 (0.99, 0.99)
Gestational length^a^			
Very preterm	20/7013 (0.003)	131/27,018 (0.0048)	0.80 (0.46, 1.40)
Moderate to late preterm	387/7013 (0.055)	1161/27,018 (0.043)	1.24 (1.08, 1.44)
Full term	6606/7013 (0.942)	25,726/27,018 (0.952)	–
Age of mother (years)	28.54 ± 5.12	28.28 ± 5.10	1.01 (1.00, 1.02)
Birthweight *z* score	–0.09 ± 1.05	–0.17 ± 1.06	1.06 (1.03, 1.10)

Data are *n* (proportion) or means ± SD

^a^Very preterm <224 days, moderate to late preterm 224–258 days, full term ≥259 days

**Table 3:** Descriptive data of maternal and newborn diagnoses among cases with childhood-onset type 1 diagnosis and controls

Variable	Cases, *n*	Controls, *n*	Cases, proportion	Controls, proportion	OR (95% CI)
Pre-eclampsia	339/14,949	1185/55,712	0.023	0.021	1.05 (0.92, 1.19)
Eclampsia	5/14,949	23/55,712	0.0003	0.0004	0.80 (0.30, 2.16)
Hypertension	74/14,949	269/55,712	0.005	0.005	1.06 (0.81, 1.38)
Jaundice, isoimmunisation and phototherapy					
Jaundice	690/14,949	2451/55,712	0.0462	0.0440	1.06 (0.97, 1.15)
Rh	12/14,949	61/55,712	0.0008	0.0011	0.77 (0.41, 1.45)
ABO	63/14,949	221/55,712	0.0042	0.0040	1.00 (0.75, 1.34)
Phototherapy	173/14,949	626/55,712	0.0116	0.0011	1.04 (0.87, 1.24)
Maternal infections					
UTI	68/14,949	199/55,712	0.0045	0.0036	1.38 (1.04, 1.83)
PROM	1236/14,949	4317/55,712	0.0827	0.0775	1.08 (1.01, 1.16)
Pyrexia and other infections during labour	46/14,949	197/55,712	0.0031	0.0035	0.84 (0.60, 1.17)
Newborn asphyxia and infections					
Intrauterine asphyxia, birth asphyxia and respiratory distress of newborn	450/14,949	1624/55,712	0.0301	0.0291	1.04 (0.92, 1.16)
Congenital pneumonia due to virus	34/14,949	120/55,712	0.0023	0.0022	1.02 (0.69, 1.51)
Neonatal aspiration syndromes	21/14,949	118/55,712	0.0014	0.0021	0.65 (0.41, 1.05)
Bacterial sepsis of newborn	160/14,949	587/55,712	0.0107	0.0105	1.02 (0.84, 1.22)

ORs are given with 95% CIs, adjusted for the case–control design

**Table 4:** Maternal and newborn diagnoses among cases with childhood-onset type 1 diagnosis and controls, stratified by age at diabetes onset

	Cases, *n*	Controls, *n*	Cases, proportion	Controls, proportion	OR (95% CI)
Age at onset: 0–4 years					
Pre-eclampsia	86/3085	282/11,580	0.028	0.024	1.11 (0.86, 1.43)
Eclampsia	0/3085	6/11,580	0	0.0005	–
Hypertension	16/3085	63/11,580	0.005	0.005	1.03 (0.59, 1.82)
Jaundice, isoimmunisation and phototherapy					
Jaundice	134/3085	513/11,580	0.0434	0.0443	0.98 (0.81, 1.20)
Rh	2/3085	17/11,580	0.0006	0.00015	0.45 (0.10, 1.96)
ABO	9/3085	51/11,580	0.0029	0.0044	0.62 (0.30, 1.28)
Phototherapy	58/3085	187/11,580	0.0188	0.0161	1.15 (0.83, 1.58)
Maternal infections					
UTI	13/3085	44/11,580	0.0042	0.0038	1.27 (0.67, 2.42)
PROM	218/3085	709/11,580	0.071	0.061	1.22 (1.03, 1.44)
Pyrexia and other infections during labour	8/3085	36/11,580	0.0026	0.0031	0.75 (0.34, 1.66)
Newborn asphyxia and infections					
Intrauterine asphyxia, birth asphyxia and respiratory distress of newborn	99/3085	376/11,580	0.032	0.032	0.98 (0.78, 1.24)
Congenital pneumonia due to virus	4/3085	35/11,580	0.0013	0.0030	0.40 (0.14, 1.14)
Neonatal aspiration syndromes	7/3085	23/11,580	0.0023	0.0020	1.14 (0.48, 2.71)
Bacterial sepsis of newborn	37/3085	104/11,580	0.0120	0.0090	1.31 (0.89, 1.93)
Age at onset: 5–9 years					
Pre-eclampsia	122/5612	444/20,800	0.0217	0.0213	0.97 (0.79, 1.20)
Eclampsia	2/5612	8/20,800	0.0004	0.0004	0.78 (0.16, 3.72)
Hypertension	29/5612	92/20,800	0.0052	0.0044	1.20 (0.78, 1.85)
Jaundice, isoimmunisation and phototherapy					
Jaundice	262/5612	871/20,800	0.0467	0.0419	1.12 (0.97, 1.30)
Rh	5/5612	18/20,800	0.0009	0.0009	1.20 (0.43, 3.33)
ABO	22/5612	77/20,800	0.0039	0.0037	0.98 (0.60, 1.60)
Phototherapy	70/5612	238/20,800	0.0125	0.0114	1.09 (0.82, 1.44)
Maternal infections					
UTI	29/5612	65/20,800	0.0052	0.0031	1.95 (1.20, 3.05)
PROM	444/5612	1517/20,800	0.0791	0.0729	1.11 (0.99, 1.25)
Pyrexia and other infections during labour	19/5612	77/20,800	0.0034	0.0037	0.84 (0.51, 1.40)
Newborn asphyxia and infections					
Intrauterine asphyxia, birth asphyxia and respiratory distress of newborn	154/5612	604/20,800	0.0274	0.0290	0.96 (0.80, 1.16)
Congenital pneumonia due to virus	11/5612	45/20,800	0.0020	0.0022	0.87 (0.44, 1.69)
Neonatal aspiration syndromes	6/5612	47/20,800	0.0011	0.0023	0.47 (0.20, 1.12)
Bacterial sepsis of newborn	54/5612	230/20,800	0.0096	0.0111	0.88 (0.65, 1.19)
Age at onset: 10–14 years					
Pre-eclampsia	131/6252	459/23,332	0.0210	0.0200	1.08 (0.88, 1.33)
Eclampsia	3/6252	9/23,332	0.0005	0.0004	1.38 (0.35, 5.49)
Hypertension	29/6252	114/23,332	0.0046	0.0049	0.96 (0.63, 1.46)
Jaundice, isoimmunisation and phototherapy					
Jaundice	294/6252	1067/23,332	0.0470	0.0457	1.03 (0.90, 1.18)
Rh	5/6252	26/23,332	0.0008	0.0011	0.72 (0.27, 1.19)
ABO	32/6252	93/23,332	0.0051	0.0040	1.22 (0.81, 1.85)
Phototherapy	45/6252	201/23,332	0.0072	0.0086	0.88 (0.62, 1.23)
Maternal infections					
UTI	26/6252	90/23,332	0.0042	0.0039	1.08 (0.69, 1.69)
PROM	574/6252	2091/23,332	0.0918	0.0896	1.03 (0.93, 1.13)
Pyrexia and other infections during labour	19/6252	84/23,332	0.0030	0.0036	0.89 (0.53, 1.49)
Newborn asphyxia and infections					
Intrauterine asphyxia, birth asphyxia and respiratory distress of newborn	197/6252	644/23,332	0.0315	0.0276	1.13 (0.96, 1.34)
Congenital pneumonia due to virus	19/6252	40/23,332	0.0030	0.0017	1.80 (1.02, 3.17)
Neonatal aspiration syndromes	8/6252	48/23,332	0.0013	0.0021	0.60 (0.28, 1.28)
Bacterial sepsis of newborn	69/6252	253/23,332	0.0110	0.0108	1.02 (0.78, 1.34)

ORs are given with 95% CIs, adjusted for the case–control design

Stratification for age at diabetes onset: 0–4 years, *n*=14,665; 5–9 years, *n*=26,412; 10–14 years, *n*=29,584

### Neonatal factors

**Fig. 1:**
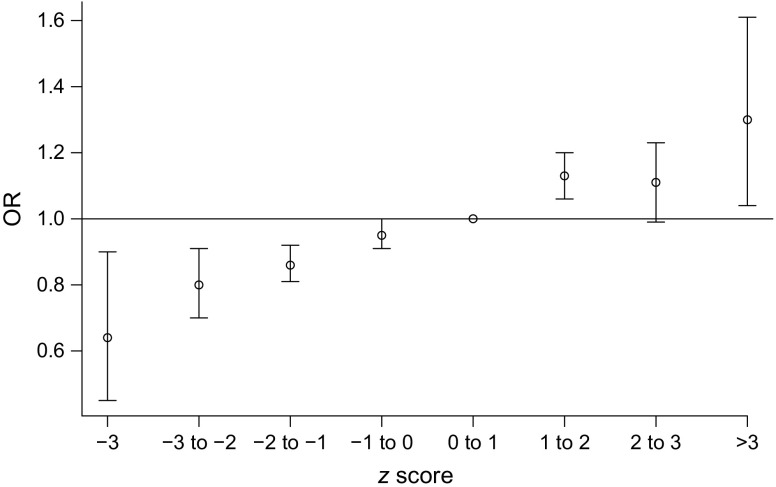
Univariate ORs and 95% CIs for the risk of childhood-onset type 1 diabetes by birthweight *z* score category with the interval 0–1 as reference. Numerical values for the 95% CIs are provided in ESM Table 4

The univariate analyses showed small but statistically significant increases in the odds for developing type 1 diabetes relative to the referent for being moderately to late premature and having a higher birthweight *z* score (Table 2). The referents for being born prematurely and birthweight *z* score were being born full term (≥259 days’ gestation) and birthweight *z* score 0–1, respectively. Being born very preterm (i.e. <32 weeks’ gestation) was associated with a lower risk of type 1 diabetes. Birthweight *z* score was positively associated with an increasing risk for type 1 diabetes (Fig. 1, ESM Table 4), with birthweight *z* scores of less than –1 associated with a decreased risk and *z* scores above 1 associated with an increased risk. The stratified results were further analysed with the RERI metric describing the amount of interaction on an additive scale. The point estimates were small and for all perinatal variables in Table 5, the 95% CIs include a 0 interaction. When analysing diagnoses, we found no significant effects of neonatal jaundice, blood-group incompatibility (rhesus factor [Rh] and ABO), asphyxia, neonatal respiratory distress, diagnoses of any infectious disease in the newborn or being treated with phototherapy (Table 3).

**Table 5:** Relative excess risk due to interaction (RERI) between sex and perinatal variables with 95% CIs

RERI with sex^a^	RERI (95% CI)
Caesarean section × sex	0.013 (–0.17, 0.18)
Birthweight × sex	0.000 (–0.0001, 0.0001)
Gestational age (days) × sex	–0.002 (–0.02, 0.03)
Gestational length^a^ × sex	
Very preterm × sex	–0.17 (–0.62, 0.34)
Moderate to late preterm × sex	–0.04 (–0.35, 0.28)
Age of mother × sex	0.002 (–0.005, 0.008)
Birthweight *z* score × sex	0.006 (–0.05, 0.06)

^a^Girls is the reference category

We further explored a possible link between neonatal jaundice or blood-group incompatibility (Rh and ABO) and risk for type 1 diabetes. However, neither stratification by age of diabetes onset (Table 4) nor by calendar-year birth cohorts (1973–1982, 1983–1992, 1993–2002 and 2003–2013) (Table 6) demonstrated any significant associations. In the earliest birth cohorts, there was a tendency towards an association with jaundice (OR 1.16; 95% CI 0.99, 1.35). The proportion of jaundice decreased from the 1973–1982 birth cohort (overall proportion 0.061, 95% CI 0.057, 0.064) to the later ones, with no overlapping of the confidence intervals [1983–1992: 0.039 (95% CI 0.036, 0.041), 1993–2002: 0.040 (95% CI 0.037, 0.042), 2003–2013: 0.041 (95% CI 0.036, 0.045)].

**Table 6:** Descriptive data for jaundice and blood-group incompatibility among cases of childhood-onset type 1 diabetes and controls, stratified according to birth cohort

Diagnosis	Cases, *n*	Controls, *n*	Cases, proportion	Controls, proportion	OR (95% CI)
Cohort 1973–1982					
Jaundice	233/3439	774/13,116	0.0678	0.0590	1.16 (0.99, 1.35)
Rh	3/3439	14/13,116	0.0009	0.0011	0.82 (0.23, 2.91)
ABO	19/3439	54/13,116	0.0055	0.0041	1.29 (0.76, 2.18)
Cohort 1983–1992					
Jaundice	170/4778	700/17,740	0.0356	0.0395	0.90 (0.76, 1.07)
Rh	3/4778	26/17,740	0.0006	0.0015	0.42 (0.13, 1.41)
ABO	22/4778	75/17,740	0.0046	0.0042	1.03 (0.63, 1.67)
Cohort 1993–2002					
Jaundice	208/5022	732/18,607	0.0414	0.0393	1.07 (0.91, 1.26)
Rh	3/5022	18/18,607	0.0006	0.0010	0.71 (0.21, 2.47)
ABO	16/5022	69/18,607	0.0032	0.0037	0.83 (0.48, 1.47)
Cohort 2003–2013					
Jaundice	79/1710	245/6249	0.0462	0.0392	1.15 (0.88, 1.51)
Rh	3/1710	3/6249	0.0018	0.0005	3.40 (0.68, 17.08)
ABO	4/1710	18/6249	0.0023	0.0029	0.68 (0.22, 2.14)

ORs are given with 95% CIs, adjusted for the case–control design

Stratification by birth cohort: 1973–1982 (*n*=16,555); 1983–1992 (*n*=22,518); 1993–2002 (*n*=23,629); 2003–2013 (*n*=7959)

### Multivariate analyses

**Fig. 2:**
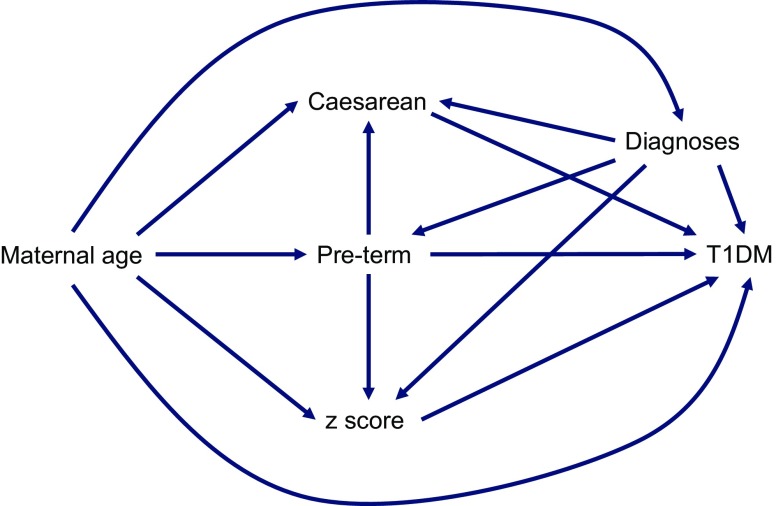
Directed acyclic graph, displaying causal models for the multivariate analyses in Table 7. ‘Diagnoses’ refers to experiencing PROM during labour or having a UTI during pregnancy. T1DM, type 1 diabetes

In the multivariate analyses, we fitted several causal models based on the directed acyclic graph (Fig. 2), which allowed us to consider different confounders. From these models, higher maternal age, being born moderately to late preterm, being born by Caesarean section, having a higher birthweight *z* score, experiencing PROM during labour and having a UTI during pregnancy were all independent risk factors for developing type 1 diabetes during childhood, whereas being born very preterm was protective (Table 7).

**Table 7:** Multivariate ORs with 95% CIs, adjusted for confounders

Model and confounders	Risk factor	OR (95% CI)	*p* value
Model 1 (maternal UTI, PROM, maternal age, preterm delivery^a^)	Caesarean section	1.08 (1.02, 1.15)	<0.01
Model 2 (maternal UTI, PROM, maternal age)	Very preterm^b^	0.54 (0.38, 0.76)	<0.001
	Moderate to late preterm^c^	1.24 (1.14, 1.35)	<0.001
Model 3 (maternal UTI, PROM, maternal age, preterm delivery^a^)	Birthweight *z* score	1.08 (1.06, 1.10)	<0.001
Model 4 (maternal age)	UTI	1.39 (1.04, 1.86)	0.025
Model 5 (maternal age)	PROM	1.08 (1.01, 1.16)	0.023

The confounders are variables that introduced spurious associations through backdoor paths in the directed acyclic graph shown in Fig. 2

^a^Gestational age at birth <259 days, ^b^gestational age at birth <224 days, ^c^gestational age at birth 224–258 days; full term ≥259 days is used as reference

### Subgroup analyses

**Fig. 3:**
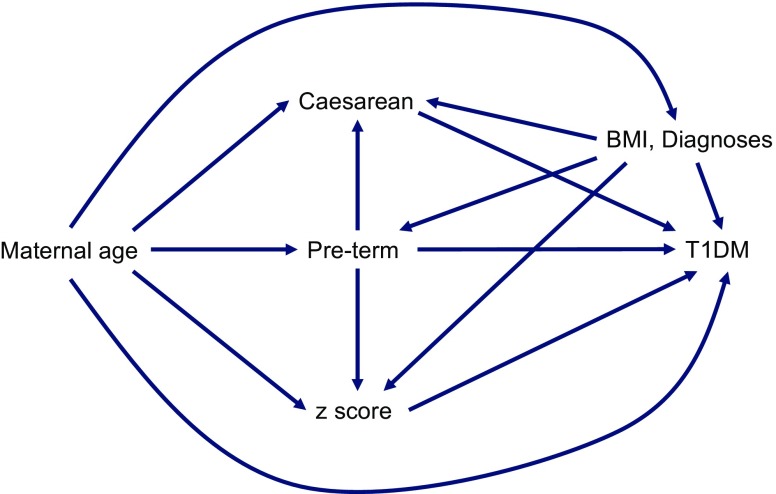
Directed acyclic graph, displaying causal models for the multivariate analyses in Table 8. ‘Diagnoses’ refers to experiencing PROM during labour or having a UTI during pregnancy. T1DM, type 1 diabetes

From 1992, the MBR has contained comprehensive data on first-trimester maternal weight and height. From this, we could calculate maternal BMI (kg/m^2^). Thus, we performed a subgroup analysis on 7196 cases and 26,579 controls with available maternal BMI data (Table 8). Here, we also fitted several causal models based on the directed acyclic graph (Fig. 3). In the subgroup multivariate analysis, we found that maternal BMI was the factor with the strongest association to later risk for type 1 diabetes with a 27% risk increase for each unit increase in BMI. Including maternal BMI in the model rendered the association with Caesarean section non-significant. However, being born moderately to late preterm, having a mother with a higher maternal age and having a higher birthweight *z* score remained independent risk factors, and being born very preterm continued to be a protective factor.

**Table 8:** Subgroup analyses: Multivariate ORs with 95% CIs, adjusted for confounders

Model and confounders	Risk factor	OR (95% CI)	*p* value
Model 1 (maternal UTI, PROM, maternal age, preterm delivery^a^, maternal BMI)	Caesarean section	1.04 (0.96, 1.15)	0.30
Model 2 (maternal UTI, PROM, maternal age, maternal BMI)	Very preterm^b^	0.64 (0.37, 0.99)	0.046
	Moderate to late preterm^c^	1.22 (1.07, 1.40)	<0.01
Model 3 (maternal UTI, PROM, maternal age, preterm delivery^a^, maternal BMI)	Birthweight *z* score	1.07 (1.04, 1.10)	<0.001
Model 4 (maternal age)	BMI: overweight^d^	1.10 (1.02, 1.19)	<0.01
	BMI: obese^d^	1.27 (1.15, 1.41)	<0.001

The confounders are variables that introduced spurious associations through backdoor paths in the directed acyclic graph shown in Fig. 3

^a^Gestational age at birth <259 days, ^b^gestational age at birth <224 days, ^c^gestational age at birth 224–258 days; full term ≥259 days is used as reference

^d^First-trimester maternal BMI: overweight, 25–30 kg/m^2^; obese, >30 kg/m^2^; <25 kg/m^2^ is used as reference

## Discussion

The present study confirms that the risk of developing type 1 diabetes during childhood is associated with a number of perinatal factors. Birthweight *z* scores of more than 1 increased the risk and birthweight *z* scores of less than –1 decreased the risk. For those with higher birthweight, these results support our earlier findings as well as those of some more recent meta-analyses. Both Harder et al [35] and Cardwell et al [15] reported a 10–17% increase in the risk for type 1 diabetes in children with birthweight >4000 g. In contrast to our findings, both meta-analyses also reported non-significant decreases in the risk for children with birthweight <2500 g. Cardwell et al [15] differentiated between cohort and case–control studies and did show that, in the cohort studies, birthweight of <2500 g was associated with a lower risk for diabetes (OR 0.79, 95% CI 0.67, 0.92), but the combined estimate was not statistically significant (OR 0.98, 95% CI 0.84, 1.13). A probable explanation for the different results may be that the meta-analyses did not account for the effect of gestational age, which was possible in the present study. In the substudy, including data from 1992–2013, we could also see that the risk for later type 1 diabetes increased in a dose-dependent fashion with increasing maternal BMI. This finding is in line with a 2015 Swedish study that reported similar correlations between higher maternal BMI and offspring type 1 diabetes [36]. However, that study included only a few perinatal risk factors, thus omitting the combined effects of some other factors on the risk of childhood type 1 diabetes. The association between high birthweight and high maternal BMI and type 1 diabetes support the so called overload or acceleration hypothesis, suggesting that a high pre- or postnatal growth rate may accelerate an ongoing beta cell destruction by nutrient overload [37, 38].

Previous studies have indicated that being born before 37 full weeks of gestation increases the risk of type 1 diabetes in the child [39]. In the present study, we show that prematurity is an independent risk factor for later type 1 diabetes, but the increased risk only applies to children born between 32 and 36 weeks of gestation, whereas birth before week 32 was protective against the later development of type 1 diabetes. Algert et al have shown similar effects of moderate to late prematurity on the risk of type 1 diabetes, but had an insufficient sample size to show significant associations for very preterm infants [40]. Prematurity has been linked to a number of alterations in immune function [41], which may have long-term consequences. Shorter gestational age has been associated with HLA categories conveying a higher risk for type 1 diabetes [42]. Other effects of premature birth include in or ex utero growth restriction, causing impaired glucose regulation later in life and changes in organ function, including endocrine feedback mechanisms or epigenetic effects [43], but how these exactly contribute to the risk of type 1 diabetes is unclear. A protective effect of very preterm birth has not been reported previously in the case of type 1 diabetes, but a recent cohort study from Sweden found that very preterm birth was associated with a lower risk for food allergies [44]. From currently available data, it is not possible to say if the protective effect against type 1 diabetes is conveyed by being very preterm per se, or by other factors leading to the preterm delivery or by the neonatal care provided to the infants.

The rate of Caesarean sections has increased worldwide over many decades, including in Sweden [45], paralleling the increase in the incidence of type 1 diabetes [7]. Caesarean section has also been associated with a higher risk for immune-mediated diseases [46], including type 1 diabetes [17], and is suggested to be linked through differences in gut microbiota composition [47, 48], although this has been recently disputed [49]. A Danish nationwide register study published in 2016 found no association between type 1 diabetes and Caesarean section [50]. In the present study, we found that birth by Caesarean section conveyed a small but statistically significant increase in the risk for type 1 diabetes. However, after allowing for maternal BMI, as we could do for those born after 1992, the association with Caesarean section disappeared. Delivery by Caesarean section is more common in older mothers [51] and in women with a higher BMI [52]. This would support the notion that it is the factors leading to Caesarean section (e.g. higher maternal age and higher maternal BMI) and not the delivery mode itself that is associated with the risk for type 1 diabetes.

When exploring the effects of other diseases during perinatal life, specifically infectious disorders, we found associations between later risk for type 1 diabetes and UTIs during pregnancy and PROM during labour, but not for any other serious infections in the mother or newborn. In animal models, perinatal antibiotic treatment of the mother has been shown to affect the risk of type 1 diabetes in the offspring, but the risk differed depending on which type of antibiotic was used [53]. It has been noted that up to 7% of women are diagnosed with a UTI, including asymptomatic bacteriuria, during pregnancy [54]. We found a very low prevalence of UTIs at only 3–4 per 1000, indicating that the birth and patient registers we used may underestimate the true prevalence, but this would only mean that our results represent a more conservative risk estimate. UTI during pregnancy has previously been associated with adverse perinatal outcomes, including an increased risk for birthweight below 2.5 kg, premature birth, maternal hypertension and pre-eclampsia and amnionitis [55]. Of these, we have shown that moderately preterm birth and high birthweight *z* score, but not maternal hypertension or pre-eclampsia, are associated with later risk for type 1 diabetes. However, the exact mechanism by which UTIs are associated with type 1 diabetes is unexplained and must be further investigated. PROM has been reported to complicate around 8% of deliveries in the USA [56], which is similar to the prevalence reported in the present study. PROM is associated with preterm birth [56], which we have shown is linked to an increased risk for type 1 diabetes.

We could not confirm earlier findings of an association between pre-eclampsia or eclampsia in the mother and type 1 diabetes in the child [9, 10]. A meta-analysis of published results [14] showed significant heterogeneity among studies and concluded that there is little evidence for a substantial effect of pre-eclampsia on the risk of type 1 diabetes. That meta-analysis, as well as the present nationwide register study, covered a substantial time span of birth cohorts. One explanation for the different findings may be that new, more active treatments during pregnancy have eradicated a possible effect of pre-eclampsia on the fetal immune system.

Neither could we confirm the repeatedly reported and more evident effect (OR between 1.5 and 3) of a relationship between later type 1 diabetes and maternal–child blood-group incompatibility or neonatal jaundice, as previously shown in Sweden and other European countries and in meta-analyses [9, 10, 14]. Again, this could be due to changing perinatal practices; for example, Rh isoimmunisation in the newborn has long been prevented by the active prenatal immune treatment of Rh-negative mothers. Moreover, we found that decreasing proportions of children had a jaundice diagnosis or ABO isoimmunisation in more recent calendar periods of birth.

The sample size of our study is larger than that of all [14–17, 35, 50] but one [39] of the recent meta-analyses, and thus allowed us to use individual-level data to thoroughly investigate the disputed relationships between perinatal factors and risk for childhood-onset type 1 diabetes. The large dataset made it possible to minimise confounding using exclusion criteria and multivariate analyses and to analyse time trends. Data were prospectively, routinely collected and stored in official registers, similarly among cases and controls. Thus, any over- or underreporting (e.g. of diagnoses) will not have led to type 1 errors, but rather to bias towards unity.

The drawbacks of a large sample size are that it may detect minor and possibly clinically less interesting associations (e.g. maternal age). A further weakness of the study is that three different ICD coding systems were used with clear difficulties in converting between them, even with lists for translation provided by Swedish National Board of Health and Welfare. Again, the same errors should be distributed equally among cases and controls since exposure data were registered long before the diabetes diagnosis. We also performed stratified analyses using the different ICD coding systems for key outcomes without finding significant differences (data not shown).

Even if the use of a large register database has great methodological advances compared with meta-analyses, the conclusions from the present data can only be drawn for Sweden. We are also aware that some of the unexpected results, such as a possible effect of UTIs in the mother, could be due to multiple comparisons and need to be further confirmed in future studies.

In conclusion*,* our comparably very large prospective and nationwide register study covering 36 years of birth cohorts confirms that perinatal factors (i.e. high birthweight *z* score, being born between 32 and 36 weeks of gestation, UTI during pregnancy and PROM during labour) are associated with childhood-onset type 1 diabetes. Some of these, such as high birthweight *z* score and high maternal BMI, lend support to the theory that a high pre- or postnatal growth rate might accelerate ongoing beta cell destruction by nutrient overload, whereas the others are mainly associated with preterm birth. Of the former, some factors, such as high maternal BMI, may be amendable with improved antenatal care.

## Electronic supplementary material

ESM (PDF 52.0 kb)

## Data Availability

The data that support the findings of this study are available from the National Board of Health and Welfare. Restrictions apply to the availability of these data, which were used under licence for the current study and are not publicly available. Data are, however, available from the authors upon reasonable request and with permission of the National Board of Health and Welfare.

## Abbreviations

MBR: Medical Birth Register
NPR: National Patient Register
PROM: Premature rupture of membranes
RERI: Relative excess risk due to interaction
Rh: Rhesus factor
SCDR: Swedish Childhood Diabetes Register
UTI: Urinary tract infection
