# Supplementary material for: Perinatal risk factors for type 1 diabetes revisited: a population-based register study
Source: Diabetologia. 2019 Apr 30;62(7):1173–84. doi: 10.1007/s00125-019-4874-5 (PMC6560018; doi:10.1007/s00125-019-4874-5)
Supplement: Supplementary file 1 — (PDF 52.0 kb) [file 125_2019_4874_MOESM1_ESM.pdf]

Electronic supplementary material (ESM)

ESM Table 1. Codes according to the World Health Organization (WHO) International Classification of Diseases (ICD) (23). The study period covered three editions, ICD 8 (until 1986), ICD 9 (1987-1996) and ICD 10 (from 1997). For phototherapy, different national codes were used until 1996, from 1997-2006 and from 2007 and onwards.

	**ICD 8**	**ICD 9**	**ICD 10**
Pre-eclampsia	63703, 63704, 63709	642E-F,H	O14
Eclampsia	63710, 63799	642 G	O15
Hypertension	63701	642, 642A-D	O10
Maternal infections			
Urinary tract	635	646G, 599A	O23, N109, N39, N30
Premature rupture of membranes	63495, 661 01-99	658B-C, 658E	O420-429
Pyrexia and other infections during labour	67299	659C-D	O752-753
Immunizations and jaundice			
Rh	77400, 77500	773A	P550
AB0	77410, 77510	773B	P551
Jaundice	77400-77499, 77500,77599 77893,77896	773A-F, 774A-G	P550-599
Neonatal asphyxia and infections			
Intrauterine asphyxia, birth asphyxia and respiratory distress of new-born	77610, 77640, 77650, 77629	768C-G, 768X, 769 A-B,X	P200-209, P210-219, P220 –229
Congenital pneumonia due to virus	4860-69	770A	P230-239
Neonatal aspiration syndromes	48501,77600	770B	P240-249
Bacterial sepsis of new-born	03800-03899, 03990	771, 771A-Z	P360-P369
	Years		
	1963-1996	1997-2006	2007-
Phototherapy	9391	V9391	DQ015

ESM Table 2. Mothers' birth countries in the study population.

Mother's country of birth	Cases	Controls
Sweden	14 323	53 499
Finland	482	1 683
Norway	72	294
Denmark	70	207
Iceland	2	29
Total	14 949	55 712

ESM Table 3. Report of missing data.

	Number of participating individuals (missing)	
Variable	Cases	Controls
Caesarean section	14 948 (<0.1%)	55 707 (<0.1%)
Birth weight	14 916 (0.2%)	55 543 (0.3%)
Gestational length	14 921 (0.2%)	55 610 (0.2%)
Age of mother	14 948 (<0.1%)	55 707 (<0.1%)
Birth weight z score	14 889 (0.4%)	55 453 (0.5%)
	Subgroup analyses (c.f. Table 7)	
	Cases	Controls
BMI	6 171 (14.2%)	22 677 (14.6%)
Caesarean section	7 196 (0%)	26 579 (0%)
Gestational length	7 192 (<0.1%)	26 557 (<0.1%)
Age of mother	7 196 (0%)	26 579 (0%)
Birth weight z score	7 176 (0.3%)	26 482 (0.4%)

ESM Table 4. Odds ratios (OR) and 95% confidence intervals (CI) of Figure 1.

Z score category	Cases	Controls	OR (95% CI)
<-3	41	230	0.64 (0.46, 0.90)
-3 to -2	295	1 343	0.79 (0.70, 0.91)
-2 to -1	1 854	7 898	0.86 (0.81, 0.92)
-1 to 0	5 018	19 143	0.96 (0.92, 1.00)
0 to 1	4 909	17 918	-
1 to 2	7 008	2 176	1.13 (1.07, 1.20)
2 to 3	1 585	480	1.11 (0.99, 1.24)
>3	328	116	1.30 (1.04, 1.62)
